# Comparing simultaneous integrated boost vs sequential boost in anal cancer patients: results of a retrospective observational study

**DOI:** 10.1186/s13014-018-1124-9

**Published:** 2018-09-10

**Authors:** Pierfrancesco Franco, Berardino De Bari, Francesca Arcadipane, Alexis Lepinoy, Manuela Ceccarelli, Gabriella Furfaro, Massimiliano Mistrangelo, Paola Cassoni, Martina Valgiusti, Alessandro Passardi, Andrea Casadei Gardini, Elisabetta Trino, Stefania Martini, Giuseppe Carlo Iorio, Andrea Evangelista, Umberto Ricardi, Gilles Créhange

**Affiliations:** 1 Department of Oncology, Radiation Oncology, University of Turin, Via Genova 3, 10126 Turin, Italy; 2 Department of Radiation Oncology, Centre Hospitalier Régional Universitaire ‘Jean Minjoz’, Besançon, France; 3 Department of Radiation Oncology, Centre ‘Paul Strauss’, Strasbourg, France; 4 Unit of Cancer Epidemiology and CPO Piedmont, AOU Citta’ della Salute e della Scienza, Turin, Italy; 5 Department of Surgical Sciences, University of Turin, Turin, Italy; 6 Department of Medical Sciences, Pathology Unit, University of Turin, Turin, Italy; 7 Department of Medical Oncology, Istituto Scientifico Romagnolo per lo Studio e la Cura dei Tumori (IRST) IRCCS, Meldola, Italy; 8 Department of Radiation Oncology, Centre ‘Georges-François-Leclerc’, Dijon, France

**Keywords:** Anal cancer radiotherapy, Concomitant radio-chemotherapy, Simultaneous integrated boost, Sequential boost, Overall treatment time

## Abstract

**Background:**

To evaluate clinical outcomes of simultaneous integrated boost (SIB) - intensity modulated radiotherapy (RT) in patients with non metastatic anal cancer compared to those of a set of patients treated with 3-dimensional conformal RT and sequential boost (SeqB).

**Methods:**

A retrospective cohort of 190 anal cancer patients treated at 3 academic centers with concurrent chemo-RT employing either SIB or SeqB was analysed. The SIB-group consisted of 87 patients, treated with 2 cycles of Mitomycin (MMC) and 5-Fluorouracil (5FU) using SIB-IMRT delivering 42-45Gy/28–30 fractions to the elective pelvic lymph nodes and 50.4-54Gy/28-30fractions to the primary tumor and involved nodes, based on pre-treatment staging. The SeqB group comprised 103 patients, treated with MMC associated to either 5FU or Capecitabine concurrent to RT with 36 Gy/20 fractions to a single volume including gross tumor, clinical nodes and elective nodal volumes and a SeqB to primary tumor and involved nodes of 23.4 Gy/13 fractions. We compared colostomy-free survival (CFS), overall survival (OS) and the cumulative incidence of colostomy for each radiation modality. Cox proportional-hazards model addressed factors influencing OS and CFS.

**Results:**

Median follow up was 34 (range 9–102) and 31 months (range 2–101) in the SIB and SeqB groups. The 1- and 2-year cumulative incidences of colostomy were 8.2% (95%CI:3.6–15.2) and 15.0% (95%CI:8.1–23.9) in the SIB group and 13.9% (95%CI: 7.8–21.8) and 18.1% (95%CI:10.8–27.0) in the SeqB group. Two-year CFS and OS were 78.1% (95%CI:67.0–85.8) and 87.5% (95%CI:77.3–93.3) in the SIB group and 73.5% (95%CI:62.6–81.7) and 85.4% (95%CI:75.5–91.6) in the SeqB, respectively. A Cox proportional hazards regression model highlighted an adjusted hazard ratio (AdjHR) of 1.18 (95%CI: 0.67–2.09;*p* = 0.560), although AdjHR for the first 24 months was 0.95 (95%CI: 0.49–1.84;*p* = 0.877) for the SIB approach.

**Conclusions:**

SIB-based RT provides similar clinical outcomes compared to SeqB-based in the treatment of patients affected with non metastatic anal cancer.

## Background

Concurrent chemo-radiation (RT-CHT) is presently considered as a standard therapeutic option in patients affected with squamous cell carcinoma of the anal canal [1, 2]. The association of pelvic radiotherapy (RT) to concomitant 5-fluorouralcil (5-FU) and mitomycin C provides high rates of complete responders (around 90%) and consistent results in terms of disease-free survival (DFS), up to 70–75% for early disease (T1-T2 tumors) and 60–65% for more advanced stages (T3-T4 or node positive tumors) at 3 years [2–4]. When RT is delivered with conventional techniques, the toxicity profile can be important, as observed in the 5-FU/MMC arm of the RTOG 9811 trial, with grade 3–4 events as high as 48% for skin and 61% for hematologic toxicity [5]. As a result, treatment breaks can be necessary with a prolongation of overall treatment time (OTT) and consequent detrimental effects on clinical outcomes [6]. Intensity-modulated radiotherapy was shown to reduce the rates of ≥ G3 acute gastrointestinal and skin toxicity and that of ≥ G2 hematologic events [7, 8]. Moreover, oncological results in terms of both local control (LC) colostomy-free (CFS) and overall survival (OS) seems promising with this treatment strategy [9, 10]. In anal cancer patients, macroscopic primary and nodal disease and elective volumes are treated to different total nominal doses [11]. In the sequential boost approach (SeqB), this can be achieved through the progressive boosting of selected target regions harboring the macroscopic disease [12]. Target volumes are progressively shrinking and boost dose is added on top of the previous sequence [13]. In the simultaneous integrated boost (SIB) strategy, a differential dose per fraction is delivered to selected sub-regions during the same treatment session, heading to different total nominal doses given to target volumes in the same number of fractions [1]. Consequently, SIB provides a reduction in OTT compared to the sequential boost approach, with a potential beneficial effect on clinical outcomes in the context of a highly repopulating tumor such as anal cancer [14]. To evaluate the potential impact of different treatment strategies on clinical outcomes, we compared data of 2 cohorts of anal cancer patients treated either with SeqB or SIB approaches, in terms of CFS as primary endpoint, after adjusting for known prognostic factors, and in terms of OS and cumulative incidence of colostomy as secondary endpoints.

## Methods

In this multi-centre retrospective observational study, we compared clinical outcomes of patients affected with anal cancer. Consecutive patients treated between 2007 and 2015 at the Radiation Oncology Departments of 3 academic Institutions were enrolled, namely a) University of Turin, Italy, b) University Hospital ‘Jean Minjoz’, Besançon, France and c) Centre ‘Georges François Leclerc’, Dijon, France. Clinical data were retrieved from local clinical databases by 2 different operators (FA and AL) and merged together on a common database used for the present analysis. The frame of the final dataset was agreed by the 2 centres. Briefly, all patients enrolled had a histologically confirmed diagnosis of anal squamous cell carcinoma (both anal canal and margin). Tumor stage was defined according to the indications of the American Joint Committee on Cancer (2002 version) and patients with clinical stage T1-T4, N0-N3, M0 were included. Patients having clinical T1 N0 tumors of the anal margin were excluded, because they were submitted to local excision. Pre-treatment clinical evaluation included complete medical history, physical examination and complete laboratory testing. Staging included a chest, abdomen and pelvis computed tomography scan (CT), a magnetic resonance imaging (MRI) of the pelvic region and positron-emission tomography (PET). A subset of patients within the SIB group, treated in center a), also received inguinal sentinel lymphnode biopsy (SLNB) for inguinal nodal staging. Patients in the SeqB, treated in centres b) and c), did not receive any SLNB procedure because of a different staging policy. Patients were followed-up according to local clinical practice and vital status was clinically updated in 2016. Written informed consent for treatment was obtained for all patients. The Ethical Review Board of each Institutional Hospital approved the present study.

### Radiotherapy characteristics

In the SeqB group, including patients treated in centres b) and c) between 2007 and 2015, the first sequence of treatment included the delivery of 36 Gy in 20 fractions (1.8 Gy daily) given over 4 weeks to the macroscopic primary and nodal tumor and elective volumes including the ischio-anal fossa and mesorectum, the pre-sacral, external and internal iliac nodes, the inguinal regions and the lower part of the common iliac lymphnodes up to the promontorium. After the first sequence a 16-day gap was planned. In the second sequence, an adjunctive dose of 23.4 Gy in 13 fractions was given to the macroscopic disease which finally received a total nominal dose of 59.4 Gy. In the SIB group, including patients treated in centre a) in the period 2007–2015, dose prescription was set according to the RTOG 0529 indications based on clinical stage at presentation [6]. Patients with cT2N0 disease were given 50.4 Gy in 28 fractions (1.8 Gy daily) to the primary anal tumor, while the elective nodal volume was prescribed 42 Gy in 28 fractions (1.5 Gy/daily). Patients presenting cT3-T4/N0-N3 disease were prescribed 54 Gy in 30 fractions (1.8 Gy daily) to the gross tumor volume, while gross nodal disease was prescribed 50.4 Gy in 30 fractions (1.68 Gy daily) if sized ≤ 3 cm or 54 Gy in 30 fractions (1.8 Gy daily) if > 3 cm. Elective nodal volume was prescribed 45 Gy in 30 fractions (1.5 Gy daily) [15, 16] . No brachytherapy boost was given to any patient in either group.

### Chemotherapy characteristics

In the SIB group, concomitant chemotherapy (CHT) consisted of 5- fluorouracil (1000 mg/m^2^/day) given as continuous infusion for 96 h (days 1–5 and 29–33) combined with mitomycin C (10 mg/m^2^) given as bolus (days 1 and 29). A total of 2 concurrent cycles were planned for each patient. In the SeqB group, at day 1 of each sequence, mitomycin C (10 mg/m^2^) was associated either with continuous infusion 5- fluorouracil (1000 mg/m^2^/day) over 96 h or capecitabine (825 mg/m^2^ bid on weekdays). Capecitabine has been used only for patients treated in centres b) and c).

### Statistical analysis

The primary study endpoint was CFS defined as the time between RT start and the date of colostomy, death, or last follow-up date on which the patient was known to be colostomy-free. Secondary endpoints were OS and cumulative incidence of colostomy. Overall survival was defined as the time between RT beginning and the date of death from any cause or last follow-up. Cumulative incidence of colostomy was calculated considering death from any cause as competing event. The number of anal cancer cases in the 3 Institutions during the study period determined the sample size. No treated patient was excluded from the present analysis and hence about 200 patients were analysed. For each cohort, the time-to-event functions of CFS and OS were estimated by the Kaplan-Meier product-limit method. Hazard Ratios (HRs) for the treatment group comparison were estimated using univariable and multivariable Cox proportional-hazards models, adjusting in the multivariable analysis for the main prognostic factors. The following variables were included in the multivariate analysis: treatment (SIB vs SeqB), gender, age as continuous variable, clinical stage (stage III vs stage I and II), and grade (G3 vs G1-G2). In order to check for the proportional-hazards assumption, we performed the Grambsch and Therneau test [17]. Although the assumption was satisfied for CFS (*p* = 0.37), we assessed the time-varying effect of treatment in the Cox model, according to 2 follow-up periods: the first 24 months after treatment and the timespan from 24 months to the end of follow-up. Considering the graphical representation of CFS, the two-year time-point was chosen because corresponding to an effect modification of treatments over time. To take into account the occurrence of death as a competing event during time, the cumulative risk of colostomy was estimated using the method described by Gooley et al. [18]. Difference between cohorts was assessed using univariable and multivariable (adjusting for the same variables used for CFS and OS) Fine & Gray models [19]. For all the endpoints, adjusted analyses were also performed including a propensity score (PS) in the regression models along with the treatment group variable. The PS for the likelihood of receiving the SIB treatment was calculated using a logistic regression model that included the same variables used in the multivariable analysis.

Because of the retrospective nature of the study, no toxicity data were retrieved apart from hematologic toxicity which was considered as reliably exploitable . Hematologic toxicity was scored according to the RTOG scoring scale.

## Results

A total of 190 patients were retrieved. The SIB group (center a) included 87 patients, while the SeqB group comprised of 103 cases (centres b and c). Detailed characteristics and comparative evaluation can be found in Table 1. Patients in the SeqB had a significant higher rate of anal margin localization (22.3% vs 16.1%; *p* < 0.0001), positive lymphnodes (48.5% vs 34.5%; *p* = 0.0015), inguinal groin involvement at diagnosis (35.9% vs 20.7%; *p* = 0.0187), and G3 differentiation (24.1% vs 16.5%; *p* = 0.0003). Borderline significant difference was found for advanced global stage (IIIA-IIIB: 54.4% vs 38.0%; *p* = 0.0730) and T-stage (T3-T4: 40.8% vs 31.0%;*p* = 0.0725) between SeqB and SIB groups (Table 1).

**Table 1:** Patient and tumor characteristics and pattern of failure

Variables	SIB group	SeqB group	Total	*p*-value
	Pts N (%)	Pts N (%)	Pts N (%)	
Sex				
*Female*	64 (73.6)	76 (73.8)	140 (73.7)	0.9722
*Male*	23 (26.4)	27 (26.2)	50 (26.3)	
Median age (range)	64 (55–70)	62 (53–77)	63 (53–77)	^a^0.4471
Subsite				
*Anal canal*	73 (83.9)	80 (77.7)	153 (80.5)	<.0001
*Anal margin*	14 (16.1)	23 (22.3)	37 (19.5)	
Histologic type				
*Basaloid*	7 (8.0)	7 (6.8)	14 (7.4)	0.7425
*Squamous cell*	80 (92.0)	96 (93.2)	176 (92.6)	
Grading				
*NA*	2 (2.4)	19 (18.4)	21 (11.1)	
*G1*	7 (8.0)	28 (27.2)	35 (18.4)	0.0003
*G2*	57 (65.5)	39 (37.9)	96 (50.5)	
*G3*	21 (24.1)	17 (16.5)	38 (20.0)	
T stage				
*T1*	5 (5.8)	4 (3.9)	9 (4.7)	0.0725
*T2*	55 (63.2)	57 (55.3)	112 (59.0)	
*T3*	23 (26.4)	25 (24.3)	48 (25.3)	
*T4*	4 (4.6)	17 (16.5)	21 (11.0)	
N stage				
*N0*	57 (65.5)	53 (51.5)	110 (57.9)	0.0015
*N1*	5 (5.8)	17 (16.5)	22 (11.6)	
*N2*	21 (24.1)	15 (14.6)	36 (18.9)	
*N3*	4 (4.6)	18 (17.4)	22 (11.6)	
Global stage				
*I*	5 (5.7)	3 (2.9)	8 (4.2)	0.0730
*II*	49 (56.3)	44 (42.7)	93 (48.9)	
*IIIA*	8 (9.3)	21 (20.4)	29 (15.3)	
*IIIB*	25 (28.7)	35 (34.0)	60 (31.6)	
Inguinal node involv.				
*NA*	0 (0%)	1 (1)	1 (0.6)	0.0187
*Yes*	18 (20.7)	37 (35.9)	55 (28.9)	
*No*	69 (79.3)	65 (63.1)	134 (70.5)	
Time biopsy-RT (days)				
*≥ 60*	62 (71.3)	5 (4.9)	67 (35.3)	0.0172
*< 60*	25 (28.7)	98 (95.1)	123 (64.7)	
OTT (days)				
*< 45*	62 (71.3)	5 (4.9)	67 (35.3)	<.0001
*≥ 45*	25 (28.7)	98 (95.1)	123 (64.7)	

^a^U-Mann Whitney Test

Legend: *SIB* simultaneous integrated boost, *SeqB* sequential boost, *pts* patients, *N*: number; involv.: Involvement, RT radiotherapy; *OTT* overall treatment time

Patients in the SIB group had a significantly higher proportion of patients with a longer time between biopsy and RT start (patient with time ≥ 60 days: 66.7% vs 49.5%; *p* = 0.0172). This was due to the inguinal SLNB procedure that was performed in some of the patients in the SIB group, which required extra time for the healing of the surgical scar. Median biopsy-RT time was 74 days (IQR: 54–98) in the SIB and 59 days (IQR: 45–73) in the SeqB groups. Median OTT (time from RT start to end) was 43 days (IQR: 42–45) in the SIB and 60 days (IQR: 58–63) in the SeqB groups. The proportion of patients with OTT ≥ 45 days was significantly higher (95.1% vs 28.7%; *p* < 0.0001) in the SeqB group. In the SeqB, 5 patients received only the first sequence of treatment because of major acute toxicity (OTT range: 28–32 days). Treatment characteristics are shown in Table 2.

**Table 2:** Treatment characteristics

Variable	N (%)
Boost approach	
*SeqB*	103 (54.3)
*SIB*	87 (45.7)
SIB dose and fractionation	
*PTV dose-tumor (Gy)*	
*54 Gy/30 fractions*	57 (65.5)
*50.4 Gy/28 fractions*	30 (34.5)
*PTV dose-positive nodes (Gy) (26 pts)*	
*50.4 Gy/30 fractions*	26 (100.0)
*PTV dose-negative nodes (Gy)*	
*45 Gy/30 fractions*	53 (60.9)
*42 Gy/28 fractions*	34 (39.1)
SeqB dose and fractionation	
*PTV dose-first sequence*	
*36 Gy/20 fractions*	103 (100)
*PTV dose-sequential boost*	
*23,4 Gy/13 fractions*	103 (100)
Chemotherapy regimens	
*5-FU + MMC × 2 cycles*	159 (83.7)
*5-FU + MMC × 1 cycle*	2 (1.0)
*Capecitabine + MMC × 2 cycles*	26 (13.7)
*Capecitabine + MMC × 1 cycle*	3 (1.6)

Legend, *SeqB* sequential boost, *SIB* simultaneous integrated boost, *PTV* planning target volume, *Gy* Gray, *pts* patients, *5-FU* 5-fluorouracil, *MMC* mitomycin C

### Pattern of failure, colostomy rates and survival

Median observation times were 34 (range 9–102) and 31 months (range 2–101) in the SIB and SeqB groups, respectively. However, out of 103 patients, 17 (16.5%) in the SeqB group were lacking of updated observation (last follow up between 1 and 4 years from analysis). In the SeqB group, the pattern of failure comprised 21 (20.4%) local, 8 (7.8%) nodal and 6 (5.8%) distant relapses. In the SIB group, local failures were 16 (18.4%), nodal 6 (6.9%) and distant 13 (14.9%). Overall, 12 colostomies (13.8%) were observed in the SIB and 17 (16.5%) in the SeqB groups, respectively. In the SIB group all colostomies were due to salvage surgery done because of local relapse. In the SeqB group all colostomies except one were due to salvage procedures. Only 1 colostomy was performed at 52 months because of functional issues.

**Fig. 1:**
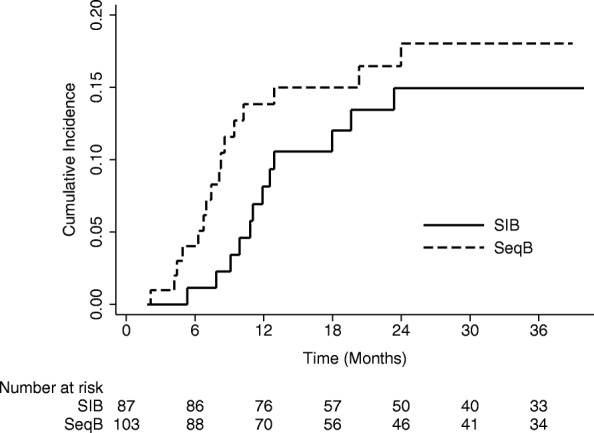
Comparative cumulative incidence of colostomy

**Fig. 2:**
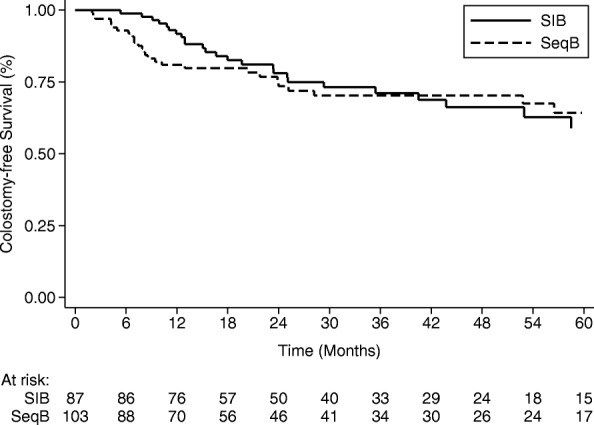
Comparative colostomy-free survival

The 1- and 2-year cumulative incidence of colostomies were 8.2% (95%CI: 3.6–15.2) and 15.0% (95%CI: 8.1–23.9) in the SIB group and 13.9% (95%CI: 7.8–21.8) and 18.1% (95%CI: 10.8–27.0) in the SeqB group (Fig. 1). Two-year CFS and OS were 78.1% (95%CI: 67.0–85.8) and 87.5% (95%CI: 77.3–93.3) in the SIB group and 73.5% (95%CI: 62.6–81.7) and 85.4% (95%CI: 75.5–91.6) in the SeqB, respectively (Fig. 2). Results from Cox proportional hazards regression models and Fine & Gray models are shown in Table 3. During the whole follow-up, SIB radiotherapy had an adjusted hazard ratio (AdjHR) of 1.18 (95%CI: 0.67–2.09,*p* = 0.560), although AdjHR for the first 24 months was 0.95 (95%CI: 0.49–1.84, *p* = 0.877) (Fig. 2). No significant differences were found between groups for OS (AdjHR = 1.51, 95%CI: 0.77–2.98, *p* = 0.235) and the cumulative incidence of colostomy (AdjHR = 0.85, 95%CI: 0.39–1.83, *p* = 0.675). Treatment comparisons adjusted using the PS approach showed results nearly identical to those of multivariable models (Table 3).

**Table 3:** Clinical factors potentially influencing colostomy-free survival

		Univariable			Multivariable			SIB vs SeqB, Propensity score adjusted		
Endpoint	Parameter	HR	95%CI	p	HR	95%CI	p	HR	95%CI	p
CSF	SIB	0.90	0.53,1.54	0.703	1.18	0.67,2.09	0.560	1.15	0.65,2.04	0.621
	SIB on the first 24 months	0.72	0.38,1.34	0.297	0.95	0.49,1.84	0.877	0.95	0.48,1.85	0.871
	Sex (M vs F)	1.64	0.93,2.90	0.087	1.60	0.90,2.84	0.109			
	Age	1.02	1.00,1.04	0.119	1.02	1.00,1.04	0.124			
	Stage III vs I-II	1.78	1.03,3.05	0.037	1.83	1.04,3.22	0.037			
	G3 vs G2-G1	0.82	0.40,1.71	0.603	0.85	0.41,1.77	0.668			
OS	SIB	1.15	0.61,2.17	0.674	1.71	0.86,3.40	0.127	1.51	0.77,2.98	0.235
	Sex (M vs F)	2.64	1.38,5.07	0.003	2.77	1.43,5.37	0.003			
	Age	1.03	1.00,1.06	0.043	1.03	1.00,1.06	0.031			
	Stage III vs I-II	2.27	1.17,4.38	0.015	2.64	1.30,5.34	0.007			
	G3 vs G2-G1	1.42	0.66,3.06	0.376	1.48	0.68,3.23	0.326			
CIC	SIB	0.71	0.34,1.47	0.352	0.87	0.41,1.85	0.713	0.85	0.39,1.83	0.675
	Sex (M vs F)	0.93	0.39,2.18	0.863	0.83	0.34,2.05	0.690			
	Age	1.00	0.98,1.03	0.765	1.00	0.98,1.02	0.878			
	Stage III vs I-II	2.09	0.99,4.38	0.052	2.13	0.97,4.70	0.061			
	G3 vs G2-G1	0.44	0.13,1.48	0.184	0.43	0.13,1.45	0.173			

Legend *HR* hazard ratio *CI* confidence interval, *M vs F male* vs *female*, *UFS* colostomy-free survival, *OS* Overall Survival, *CIC* Cumulative Incidence of colostomy

No difference (*p* = 0.65) in terms of ≥G3 hematologic toxicity was found between the SeqB group (22%) and the SIB group (26%).

## Discussion

Combined RT-CHT in a concomitant setting is the standard of cancer for anal cancer patients [11]. In Europe, split-course high-dose RT was mostly chosen as a treatment option following established seminal works [20]. A treatment gap was planned between the first large-field phase and the second boost phase on the macroscopic disease. The gap was intended to allow for the resolution of acute skin and mucosa toxicity and for tumor response assessment to better tailor the subsequent overdosage on the residual disease [7]. This approach was set by 2 randomized phase III trials namely the ACT I and EORTC 22861 trials, which demonstrated the benefit of adding CHT over RT alone, in terms of local control, sphincter preservation rate and overall survival [21, 22]. In these trials, RT was given employing 2 treatment sequences delivered sequentially. In the EORTC 22861 trial, 45 Gy over 5 weeks were given using conventional fractionation to the whole pelvis followed, after a 6-week interval, by a boost dose modulated according to treatment response (20 Gy to partial and 15 Gy to complete responders) and delivered thorough photons, electrons or ^192^Ir implants [21]. Concomitant CHT (continuously infused 5-FU and bolus MMC) was given only during the first treatment sequence [21]. Similarly, in the ACT I trial, the first phase was made up of 45 Gy in 20 or 25 fraction over 4 to 5 weeks, while the boost (given to complete or > 50% partial responders) employed 15 Gy in 6 fractions with photons or electrons or 25 Gy given at 10 Gy per day given with iridium implants [22]. Mitomycin C was given at the beginning of the first phase and 5-FU at the beginning and end of it. In the US, clinical researches mostly employed a moderate dose and continuously delivered RT course associated to concurrent chemotherapy as in the RTOG 8704-ECOG 1289 trial, in which patients were treated up to 45–50.4 Gy, with field reduction at 30.6–36 Gy, with concurrent 5-FU ± MMC for 2 cycles [23]. Based on the observation that accelerated tumor cell repopulation occurs during RT and may have detrimental effects on clinical outcomes, clinical research started to shorten treatment regimens to decrease overall treatment time [24]. For example the EORTC 22953 phase II trial, investigated the feasibility and toxicity profile of a reduction in the gap period between the whole pelvis phase and the boost to 2 weeks, together with the intensification of the CHT regimen [25]. The compliance to therapy in terms of delivered dose and treatment duration was 93%, with a complete response rate of 90.7% [25]. More recently, SeqB approaches were delivered with no pre-planned interruptions as in the ACT II trial and represent, nowadays, the standard treatment strategy [2]. Neverthelss, compared to the SeqB approach, SIB potentially allows for a further reduction in the OTT, because treatment fractions are continuously delivered and different daily doses to target volumes are given in the same number of fractions [1, 10]. In the attempt to investigate whether SIB may provide a therapeutic gain, we analyzed 2 cohorts of anal cancer patients treated with SIB and SeqB approaches. To the best of our knowledge, this is the first study comparing these 2 treatment strategies in terms of CFS in anal cancer patients. Median follow up was similar for the 2 groups (34 vs 31 months for SIB and SeqB). Focusing on patients characteristics, the 2 cohorts were different since the SeqB group had a significant higher proportion of patients with tumor of the anal margin (22.3% vs 16.1%; *p* < 0.0001), with positive nodes (48.5% vs 34.5%; *p* = 0.0015), inguinal groin localization (35.9% vs 20.7%; *p* = 0.0187), and G3 differentiation (24.1% vs 16.5%; *p* = 0.0003). Borderline significant difference was found for advanced stage (IIIA-IIIB: 54.4% vs 38.0%;*p* = 0.0730). Conversely, patients in the SIB group had a longer time between biopsy and RT start (patient with time ≥ 60 days: 66.7% vs 49.5%; *p* = 0.0172). Median OTTs were 43 and 60 in the SIB and SeqB groups, respectively, due to the different approaches in delivering radiation. Local relapse rate was slightly higher for patients submitted to sequential boost (SeqB: 20.4% vs SIB: 18.4%), as were regional failures (SeqB: 7.8% vs SIB: 6.9%). A higher percentage of distant metastasis was observed in the SIB group (14.9%) compared to the SeqB group (5.8%), even if baseline patients characteristics were more favorable for these patients. A slightly, but non significant, higher colostomy rate was seen in the SeqB group (16.5% vs 13.8%). Noteworthy, in this group, one surgical procedure was performed because of functional issues in this cohort.

After adjusting for known prognostic factors, the effect of undergoing different treatments highlighted an AdjHR with respect to 2-year CFS for the first 24 months of 0.95 (95%CI:0.49–1.84, *p* = 0.877), with an HR supposedly favoring the SIB approach, even if with a confidence interval containing the value 1 and thus not considerable as statistically significant (Fig. 1). We focused on the first 2 years because, for 17 out of 103 patients in the SeqB group, we could not retrieve an updated follow up (last observation time between 1 and 4 years from analysis), which could potentially affect the CFS rates as death from any cause is considered as an event. Interestingly, the cumulative incidence of colostomies at 1 and 2 years was higher in SeqB group (13.9%;95%CI:7.8–21.8 and 18.1%;95%CI: 10.8–27.0, respectively) compared to those of the SIB group (8.2%;95%CI:3.6–15.2 and 15.0%;95%CI: 8.1–23.9, respectively). We can hypothesize that it may suggest an explanation for the observed HR with respect to 2-year CFS when considering the SIB group. This observation may potentially be ascribed to the reduction in OTT given by the SIB strategy, but others clinical factors related to both patient and tumor that were not taken into account in the present analysis might have a consistent role. Given the retrospective nature of the present study and the unbalancement in terms of clinical characteristics between the 2 groups, we could not assess the superiority of one approach over the other nor the equivalence between them. Nevertheless, the clinical results reported within the SIB group are reassuring and strongly suggest the equipoise between the two strategies. Confirmatory prospective randomized trials would be need to prove this hypothesis. It is also interesting to observe that, albeit having a higher proportion of patients with high risk features in the SeqB group, no significant outcome differences were observed. A potential role of stage migration due to the use of inguinal SLNB in the SIB group can be pointed out to partially explain this finding. Several series have shown the detrimental effect of a longer OTT in anal cancer patients submitted to concurrent RT-CHT. Weber et al. have shown the a gap longer that 37.5 days had a significant effect on clinical outcomes in patient treated with split- course radiation [7]. In their series patients with a longer gap had a 75% loco-regional control rate compared to 92.3% for patients with a shorter gap [7]. Graf et al. found that OTT > 41 days significantly affected 5-year local control in anal cancer patients treated with RT-CT, as the rate was 58% for patients having OTT > 41 days and 79% for those with a OTT < 41 days (*p* = 0.04) [14]. This correlation was found regardless of the treatment approach used, either split-course or continuously delivered radiation. Pooled data analysis of patients enrolled in the RTOG 8704 and RTOG 9811 trials have shown a correlation between OTT and local failure, loco-regional failure, colostomy failure and time to failure, but not with CFS or OS [26]. In the RTOG 92–08 trial, dose escalation up to 59.9 Gy concurrent to 5-FU and MMC was investigated with a mandatory 2-week gap after 36 Gy [27]. Clinical outcomes were poor even with a higher dose delivered, suggesting a detrimental effect even of a short gap. Comparison between patients with similar characteristics having a 2-week gap (RTOG 9208) and those without (RTOG 8704) highlighted poorer results for patients with a longer gap [25]. The aforementioned clinical data are explained from a radiobiological point of view by accelerated repopulation that occurs after irradiation and may lead to a loss in tumor control of 1–2% and of 0.4–0.6 Gy for each day of OTT extension [28, 29]. Split-course studies demonstrated that a gap longer than 15 days may consistently affect clinical outcomes [28, 29]. Our study has some limitations including its retrospective nature, the missing of updated follow up for some patients in the SeqB cohort, the lack of the chance to adjust results for all tumor- and patient-related factors potentially affecting clinical outcomes and the maximum reliability of the analysis for the first 24 months of follow up only. Moreover the sample size determination might not be adequate enough to detect a small difference in clinical outcomes. Hence, our data cannot be considered conclusive. Another important limit is the lack of robust data on the toxicity profile that did not allow us to compare the 2 approaches with respect to this endpoint. The only toxicity endpoint analysed in the present study was hematologic toxicity which did not show any difference in the 2 groups with respect to ≥ G3 events. Toxicity data from the RTOG 0529 trial showed a reduction of skin, gastro-intestinal and hematologic toxicity for patients treated with dose-painted IMRT compared to standard treatment. The same treatment schedule was adopted in the SIB group of the present study.

Nevertheless, the comparison between SeqB and SIB approaches seems to suggest that SIB is non-inferior to SeqB in terms of probability of being alive without a colostomy in anal cancer patients, and it could be a valid approach in this clinical setting. A potential advantage in terms of CFS can be hypothesized for SIB due to the shortening in OTT, but this possible finding need to be confirmed with more robust and prospective data.

## Conclusions

The present study is the first to report on comparative outcomes of anal cancer patients treated with RT-CHT employing a SIB strategy compared to a SeqB approach. Given its multicenter retrospective frame, potential selection biases due to the different patient and treatment characteristics between the 2 cohorts can be outlined. Different approaches in terms of patient care and follow up modalities, including time to event evaluation, may have had an influence on outcomes. Patients were given different doses to the prophylactic nodal volumes (36 Gy vs 42/45 Gy for the SeqB and SIB group, respectively), even if comparable in terms of biologically equivalent dose since given with diverse dose per fractions (1.8 Gy vs 1.5 Gy daily). Dose to the macroscopic tumor was different (59.4 Gy vs 54 Gy) as delivery technique (3DCRT vs IMRT) for the SeqB and SIB group, respectively. Patients in the SeqB group had, on average, a more advanced stage of disease presentation, while patients in the SeqB had a longer time between biopsy and chemo-radiation start. However, those unbalances were properly adjusted during the analysis to obtain an AdjHR depending mainly on treatment strategy (SIB vs SeqB). One of the pitfall of the study was the lack of toxicity data. We decided not to report on toxicity because of the retrospective nature of the study and the consequent lack of reliability for these outcomes. Thus, we decided to focus on CFS, OS and cumulative incidence of colostomy, since death and colostomy were deemed events whose detection was thought to be sufficiently reliable. With these robust clinical endpoints as comparative parameters, both SIB and SeqB approaches proved to be effective treatment strategies to be included in the combined modality therapy of non-metastatic anal cancer patients.

## Abbreviations

5-FU: 5-fluorouracil
AdjHR: Adjusted hazard ratio
CFS: Colostomy-free survival
CT: Computed tomography
DFS: Disease-free survival
HRs: Hazard ratios
LC: Local control
MRI: Magnetic resonance imaging
OS: Overall survival
OTT: Overall treatment time
PET: Positron emission tomography
PS: Propensity score
RT: Radiotherapy
RT-CHT: Concurrent chemo-radiation
SeqB: Sequential boost
SIB: Simultaneous integrated boost
SLNB: Sentinel lymphnode biopsy
